# Anti-epileptic drugs and prostate cancer-specific mortality compared to non-users of anti-epileptic drugs in the Finnish Randomized Study of Screening for Prostate Cancer

**DOI:** 10.1038/s41416-022-01817-3

**Published:** 2022-05-03

**Authors:** Jukka K. Salminen, Aino Mehtola, Kirsi Talala, Kimmo Taari, Jussi Mäkinen, Jukka Peltola, Teuvo L. J. Tammela, Anssi Auvinen, Teemu J. Murtola

**Affiliations:** 1 Tampere University, Faculty of Medicine and Health Technology, Tampere, Finland; 2 Finnish Cancer Registry, Helsinki, Finland; 3 Department of Urology, Helsinki University and Helsinki University Hospital, Helsinki, Finland; 4 Lapland Central Hospital, Department of Neurology, Rovaniemi, Finland; 5 Tampere University Hospital, Department of Neurology, Tampere, Finland; 6 Tampere University Hospital, Department of Urology, Tampere, Finland; 7 Tampere University, Faculty of Social Sciences, Tampere, Finland

**Keywords:** Epidemiology, Prostate cancer, Prostate cancer

## Abstract

**Background:**

Drugs with histone deacetylase inhibitory (HDACi) properties have shown to decrease prostate cancer (PCa) cell growth in vitro.

**Methods:**

A cohort of 9261 PCa cases from the Finnish Randomized Study of Screening for Prostate Cancer (FinRSPC) was used to evaluate prostate cancer-specific mortality in men using anti-epileptic drugs (AEDs). A national subscription database was used to obtain information on medication use. Cox regression with AED use as a time-dependent variable was used to analyse prostate cancer mortality in men using AEDs compared to non-users, and in men using HDACi AEDs compared to users of other AEDs. The analysis was adjusted for age, screening trial arm, PCa risk group, primary treatment of PCa, Charlson co-morbidity score and concomitant use of other drugs.

**Results:**

The use of AEDs, in general, was associated with an increased risk of PCa death. The use of HDACi AEDs was not significantly associated with decreased PCa mortality compared to use of other AEDs (HR 0.61, 95% CI 0.31–1.23).

**Conclusions:**

AED usage is associated with elevated PCa mortality compared to non-users, likely reflecting the differences between men with epilepsy and those without. No benefit was observed from HDACi drugs compared to other AEDs.

## Background

Prostate cancer (PCa) is the second most common malignancy worldwide in men and one of the most common causes of cancer death [1]. Valproic acid, an anti-epileptic drug (AED) with histone deacetylase inhibitory (HDACi) properties decreases prostate cancer cell proliferation in vitro and tumour volume in vivo [2–5]. It is also reported to have an anti-angiogenic effect on prostate cancer cells [6, 7]. Of other AEDs, carbamazepine and topiramate have HDACi properties [8–10].

Previous studies of prostate cancer risk in users of AEDs have shown conflicting results. Valproic acid users showed a non-significantly increased prostate cancer risk in a UK cohort study [11]. Valproic acid, carbamazepine, oxcarbazepine, lamotrigine and levetiracetam may decrease serum levels of prostate-specific antigen (PSA) [12]. No association between prostate cancer risk and long-term valproic acid usage was found in two previous studies [13, 14]. We found a decreased risk of PCa associated with the usage of valproic acid, carbamazepine and phenobarbital in a population-based case-control study [15]. To our knowledge, no studies have assessed prostate cancer mortality in users of AEDs.

Epilepsy is associated with increased cancer mortality overall. However, no studies that we know of have examined prostate cancer mortality in people with epilepsy. [16–18] There have been reports of cancer incidence in users of AEDs, but there is no consensus whether anti-epileptic drugs either promote cancer or protect from it [19–21].

We examined the association between AED use and prostate cancer-specific mortality with a focus on HDAC inhibitors among men in the Finnish Randomized Study of Screening for Prostate Cancer (FinRSPC) during 1996–2015.

## Methods

### The study cohort

The FinRSPC included 80,458 men residing in the metropolitan areas of Helsinki and Tampere [22]. All men aged 55, 59, 63 and 67 in the target population were identified from the Population Register in Finland annually from 1996 to 1999. Prevalent prostate cancer cases were excluded and after that men were randomly assigned into two groups: the screening arm (32,000 men) and the control arm (48,458 men). Men in the screening arm were invited to screening with prostate-specific antigen (PSA) every 4 years until the age of 71 years, excluding men who had been diagnosed with PCa, emigrated or died.

PCa cases in both arms were identified from the Finnish Cancer Registry, which covers practically all cancer cases in Finland [23]. The clinical information on Gleason grade (available for 97.3% of cases), stage (97.7%) and serum PSA concentration at diagnosis was acquired from the medical records. A total of 9261 PCa cases were diagnosed until the end of 2015. These cases formed our study cohort.

Causes of death were obtained from the death certificate database of Statistics Finland (authorisation number TK-53-1330-18). Statistics Finland separately records primary, secondary and immediate causes of death as ICD-10 codes. Our analysis included deaths until the end of 2015. Deaths with ICD-10 code C61 recorded as the primary cause of death were considered prostate cancer deaths. The accuracy of PCa death in the death certificate database has previously been validated by the FinRSPC cause of death committee [22].

Statistics Finland provided information on socioeconomic factors, such as marital and occupational status. Data on occupational status was available for 7344 men (79.3% of the study cohort) and on marital status for 7543 men (81.4% of the study cohort). Information on BMI was available for 985 men (10.6% of the study cohort), who responded to a questionnaire mailed along with the third-round FinRSPC screening invitations.

### Information on medication use

The study cohort was linked to the national prescription database of the Social Insurance Institution (SII) of Finland using personal identification numbers to obtain information on AED purchases during 1995–2015. Additionally, information on the usage of statins, anti-diabetic medication, anti-hypertensive medication, NSAIDs and aspirin were obtained as potential confounders from the database. Information on medication use was obtained for 8857 men in the study population (95.6 %).

The SII is a governmental agency providing reimbursements for the costs of prescription medication [24]. Reimbursement is available for all Finnish residents, usually obtained as a price subsidy at purchase at the pharmacy. For every reimbursed purchase, the date, number of packages, dose and number of doses of the purchase are entered into the database. Drugs dispensed to hospital inpatients or in other ways institutionalised patients are not covered by the prescription database. All AEDs were available through a physician’s prescription only and thus purchases are comprehensively recorded by the database.

In Finland, all patients with epilepsy have a right to 50–100% reimbursement for AEDs. Getting 100% reimbursement requires the confirmed diagnosis of epilepsy based on clinical neurological assessment, electroclinical findings and neuroimaging interpreted by neurologists. Fifty percent reimbursement is available with a prescription to all Finnish citizens even without a diagnosis of epilepsy. The SII database was used to identify all participants with AED use regardless of imbursement level. A subgroup of men with 100% reimbursement was used for a separate analysis. Data of 100% reimbursement was only available for the years 1995–2009.

The anti-epileptic drugs licensed in Finland during 1995–2015 were ethosuximide, phenobarbital, phenytoin, gabapentin, carbamazepine, clonazepam, lamotrigine, levetiracetam, oxcarbazepine, pregabalin, primidone, tiagabine, topiramate, zonisamide, lacosamide, valproic acid and vigabatrin. The drugs were identified from the SII prescription data based on drug-specific ATC codes. Clonazepam, phenobarbital, pregabalin and primidone were excluded from the analysis either because of their common usage for other indications than epilepsy (clonazepam and pregabalin) or because of a very small number of users (less than 10 men) in the study population during the follow-up (phenobarbital and primidone).

The users of carbamazepine, topiramate, oxcarbazepine and valproic acid were classified as users of HDACi medication and users of other AEDs as non-HDACi users. Oxcarbazepine was considered a HDAC inhibitor due to its close pharmacological similarity to carbamazepine.

Over-the-counter use of NSAIDs and aspirin is not recorded by the prescription database. Therefore, a proportion of participants in the FinRSPC were mailed a survey in 2004–2008 measuring prescription-free usage of NSAIDs and aspirin [16]. The survey was sent along with the third-round screening invitations. This information was available for 992 out of 9261 (10.7%) men in the cohort.

### Statistical analysis

Cox regression was used to estimate hazard ratios (HR) and their 95% confidence intervals (95% CI) for prostate cancer death and death due to any cause according to the usage of AEDs. Additional comparisons were performed between users of HDACi AEDs and users of other AEDs. We also analysed the hazard of prostate cancer death in men using HDACi AEDs compared to non-users of any anti-epileptic medication. Valproic acid (VPA) was also analysed separately relative to non-HDACi, because in vitro studies on the topic have almost exclusively evaluated the effect of VPA on cancer growth. Follow-up began at the date of prostate cancer diagnosis and continued until death, emigration or 31.12.2015, whichever occurred first.

Cox regression was adjusted for age and in the multivariable analyses also for simultaneous usage of other drugs (NSAIDs, aspirin, anti-diabetic drugs, anti-hypertensive drugs and statins), screening trial arm, Charlson co-morbidity score, prostate cancer risk group and primary treatment of PCa. Prostate cancer risk groups were categorised as low, medium or high. A low-risk PCa was required to fill all the following criteria: Gleason score 6 or below, stage T1-2 and PSA under 10. Medium-risk PCa had at least one of the following characteristics: Gleason 7, stage T3 or PSA 10–20. High-risk PCa was defined as having one of the following properties: Gleason score 8–10, stage T4, metastases or PSA over 20. Primary treatment options were active surveillance/watchful waiting, radical prostatectomy, radiation treatment, endocrine treatment or palliative treatment.

AED usage before and after prostate cancer diagnosis was analysed separately. Pre-diagnostic use was analysed as a fixed baseline variable. Men with any anti-epileptic drug use before the diagnosis were categorised as ever-users, those with no purchases as never-users. Usage after diagnosis was included in the Cox regression as a time-dependent variable. Medication usage status was updated yearly after the diagnosis according to recorded purchases. Men who were not anti-epileptic drug users at the time of PCa diagnosis were considered non-users until the year of the first purchase. After the first recorded purchase of AED, the status remained as a user for each year with registered purchases of AEDs. If AED purchases ceased, the status returned to non-user. The analysis included two time-dependent variables, one for the HDACi AED use and another for the use of other AEDs. AED users were categorised as HDACi users each year when HDACi drug purchases were recorded. For years with purchases for only non-HDACi AEDs they changed the category to non-HDACi users. The category was allowed to change as many times as indicated by drug purchases.

To assess AED use for epilepsy only and not for other AED use indications (such as migraine and chronic pain), we performed separate analyses including only AED users with confirmed epilepsy, i.e. men with 100% reimbursement for AEDs.

The amount of medication used between different drugs was standardised by dividing the yearly mg amount of drugs purchased with the drug-specific Defined Daily Dose (DDD) listed by the World Health Organization [17]. Yearly DDD amounts were added together to obtain the total cumulative amount of anti-epileptic drug use. Length of usage was measured as the number of years with recorded purchases regardless of the amounts, and the average intensity of usage was calculated by dividing the cumulative amount of DDDs by the years of usage. For pre-diagnostic use, the cumulative amount, duration and intensity of AED use were analysed as fixed baseline variables. For post-diagnostic use, these were time-dependent variables, cumulating over the follow-up with continued yearly purchases. For men discontinuing anti-epileptic drug use, the cumulative amount stayed at the level reached before the discontinuation.

To evaluate delayed risk associations between AED use and prostate cancer mortality and to minimise the impact of protopathic bias, we performed lag-time analyses where exposure occurred 1–3 years before the outcome was censored. For instance, outcomes occurring in the year 2002 by AED use in 2001 were analysed in the 1-year lag-time analysis.

We performed a sensitivity analysis using a new-user design, which excluded all AED users who started usage before PCa diagnosis (*N* = 296). Also, a sensitivity analysis taking account only the use of AEDs 2 years prior to PCa diagnosis was performed.

To assess effect modification by the background variables (age, trial arm, primary treatment for PCa, PCA risk group), the analysis was stratified according to the background variables and interaction with medication usage was evaluated by adding an interaction term to the Cox regression model. Improvement in model fit was assessed using likelihood ratio tests.

Cox regression analyses were performed using IBM SPSS statistical software version 25 (Chicago, IL, USA).

### Role of the funding source

External funding for the study was non-restrictive: funders had no role in the design of the study, the collection, analysis or interpretation of the data, the writing of the article or in the decision to publish the results. The corresponding author confirms that he had full access to all the data in the study and had final responsibility for the decision to submit it for publication.

## Results

### Population characteristics

Of the total 9261 PCa cases, 8521 (92%) had never used AEDs, while 740 men with PCa (8.0%) had used AEDs and of them, 454 (5.0%) had used HDACi AEDs (Table 1). Of the 740 men having used AEDs during 1995–2015, 603 men (81%) had used only 1 AED during this period. Of men having used only 1 AED, 330 (55%) had used HDACi AEDs and 273 (45%) non-HDACi AEDs. 19% of the men having used AEDs (*N* = 137) during the study period had used 2 or more AEDs, with 24 of them having used only HDACi AEDs and 13 having used only non-HDACi AEDs. One hundred AED users (14%) had used both HDACi and non-HDACi AEDs during 1995–2015.

**Table 1:** Population characteristics.

	Anti-epileptic drug use		
	None	Any	HDAC inhibitor use
N of prostate cancer cases	8548	740	454
N of deaths	2639 (30.9%)	305 (42.8%)	205 (48.0%)
N of prostate cancer deaths	848 (9.9%)	72 (10.1%)	40 (9.4%)
N of (1) low	(1) 2903 (35.9%)	(1) 229 (33.3%)	(1) 133 (32.7%)
(2) medium	(2) 2843 (35.2%)	(2) 276 (40.2%)	(2) 166 (40.8%)
(3) high-risk PCa	(3) 2330 (28.9%)	(3) 182 (26.5%)	(3) 108 (26.5%)
Median age at diagnosis	69	69	69
Screening arm	3498 (40.9%)	296 (41.5%)	173 (40.5%)
Use of other drugs:			
anti-diabetic drugs; *n* (%)	1614 (19.8%)	120 (17.5%)	63 (15.4%)
statins; *n* (%)	4581 (56.1%)	402 (58.7%)	228 (55.6%)
anti-hypertensive drugs; *n* (%)	6583 (80.6%)	596 (87.0%)	349 (85.1%)
aspirin; *n* (%)	1298 (15.9%)	141 (20.6%)	88 (21.5%)
NSAIDs; *n* (%)	7494 (91.7%)	654 (95.5%)	386 (94.1%)
Primary treatment			
Active surveillance/watchful waiting	1714 (20.4%)	154 (21.6%)	96 (22.5%)
Radical prostatectomy	1872 (21.9%)	118 (16.5%)	62 (14.5%)
Radiation therapy	3285 (38.4%)	279 (39.1%)	175 (41.0%)
Endocrine therapy	1499 (17.5%)	149 (20.9%)	82 (19.2%)
Palliative	7 (0.1%)	0 (0.0%)	0 (0.0%)
Other	144 (1.7%)	13 (1.8%)	12 (2.8%)

Study cohort of 9261 prostate cancer cases from the FinRSPC.

The median age at PCa diagnosis was 69 in both men using AEDs and men without AED use. The use of anti-hypertensive drugs, aspirin and NSAID was more common in users of AEDs. Radical prostatectomy was less often the first treatment in men using AEDs. In the subgroup of 102 men with 100% reimbursement for AEDs 54 died during the follow-up, 11 due to PCa.

### Prostate cancer mortality compared to non-users of AEDs

A total of 848 PCa deaths were recorded among men not using AEDs, corresponding to 99 PCa deaths/1000 PCa cases. Users of AEDs had a total of 72 PCa deaths, 101 PCa deaths/1000 PCa cases. Both AED use before prostate cancer diagnosis (multivariable adjusted HR 1.39, 95% CI 0.98–1.99) and after prostate cancer diagnosis (HR 1.42, 95% CI 1.00–2.03) were associated with increased prostate cancer mortality compared to non-users of AEDs (Table 2). The intensity of AED use had no significant trend with PCa mortality. In an analysis limited to AED users with confirmed epilepsy, no difference in prostate cancer mortality was seen compared to non-users of AEDs (multivariable adjusted HR 0.78, 95% CI 0.37–1.63).

**Table 2:** Risk of prostate cancer death by anti-epileptic drug use.

Risk of prostate cancer death							
AED use before diagnosis				AED use after diagnosis			
	Number of PCa deaths/PCa cases	HR (95% CI)_age adjusted_	${{{{{\mathbf{HR}}}}}} ({{{{{\mathbf{95\%}}}}}} {{{{{\mathbf{CI}}}}}})_{{{{{{\mathbf{multivariable}}}}}}\; {{{{{\mathbf{adjusted}}}}}}^{\mathbf a}}$		Number of PCa deaths/PCa cases	HR (95% CI)_age adjusted_	${{{{{\mathbf{HR}}}}}} ({{{{{\mathbf{95\%}}}}}} {{{{{\mathbf{CI}}}}}})_{{{{{{\mathbf{multivariable}}}}}}\; {{{{{\mathbf{adjusted}}}}}}^{\mathbf a}}$
No AED use	883/8965	Ref.	Ref.	No AED use	861/8665	Ref.	Ref.
Ever use of AEDs	37/296	1.71 (1.23–2.38)	1.39 (0.98–1.99)	Ever use of AEDs	59/596	1.72 (1.21–2.43)	1.42 (1.00–2.03)
**Intensity of usage (DDDs per year)**							
1st tertile	10/103	1.30 (0.70–2.42)	1.92 (1.02–3.59)	1st tertile	21/183	4.39 (2.42–7.96)	4.52 (2.48–8.20)
2nd tertile	12/94	1.64 (0.93–2.90)	0.87 (0.45–1.67)	2nd tertile	22/216	1.76 (0.94–3.28)	1.22 (0.63–2.35)
3rd tertile	15/99	2.27 (1.36–3.78)	1.77 (1.02–3.08)	3rd tertile	16/197	1.09 (0.62–1.92)	0.95 (0.54–1.68)
P for trend by tertile		0.002	0.085	P for trend by tertile		0.09	0.45

Study cohort of 9261 prostate cancer cases from the FinRSPC.

^a^Calculated with Cox regression adjusted for age, simultaneous usage of other drugs (NSAIDs, aspirin, anti-diabetic drugs, anti-hypertensive drugs and statins), FinRSPC screening trial arm, Charlson co-morbidity score, prostate cancer risk group and primary treatment of PCa.

### Prostate cancer mortality by HDAC use

Forty users of HDACi AEDs died from PCa, 93 PCa deaths/1000 PCa cases. Post-diagnosis use of HDACi AEDs was not significantly associated with lower prostate cancer mortality compared to the usage of other AEDs (multivariable adjusted HR 0.61, 95% CI 0.31–1.23) (Table 3). However, prostate cancer mortality correlated inversely with an average intensity of HDACi drug usage, with a significant linear trend by intensity tertiles. When comparing the use of HDACi AEDs before diagnosis to the use of other AEDs, no difference in prostate cancer mortality was seen. Post-diagnostic users of HDACi AEDs had no statistically significant excess PCa mortality compared to men not using AEDs (HR 1.15, 95% CI 0.71–1.87) (Supplementary Table 1). The use of valproic acid showed no difference in PCa mortality when compared to the use of non-HDACi AEDs or non-users of AEDs (Supplementary Table 3). When including only men with confirmed epilepsy, no difference in prostate cancer mortality was seen among users of HDACi AEDs relative to users of other AEDs (multivariable adjusted HR 2.38, 95% CI 0.29–19.84).

**Table 3:** Risk of prostate cancer death by use of anti-epileptic drugs with HDAC inhibitory properties compared to usage of other anti-epileptic drugs.

Risk of prostate cancer death							
	HDACi AED use before diagnosis				HDAC use after diagnosis		
	Number of PCa deaths/PCa cases	HR (95% CI)_age adjusted_	${{{{{\mathbf{HR}}}}}}\,({{{{{\mathbf{95\%}}}}}} {{{{{\mathbf{CI}}}}}})_{{{{{{\mathbf{multivariable}}}}}}\; {{{{{\mathbf{adjusted}}}}}}^{\mathbf a}}$		Number of PCa deaths/PCa cases	HR (95% CI)_age adjusted_	${{{{{\mathbf{HR}}}}}} ({{{{{\mathbf{95\%}}}}}} {{{{{\mathbf{CI}}}}}})_{{{{{{\mathbf{multivariable}}}}}}\; {{{{{\mathbf{adjusted}}}}}}^{\mathbf a}}$
Ever use of non-HDACi AED	8/112	Ref.	Ref.	Ever use of non-HDACi AED	37/318	Ref.	Ref.
Ever use of HDACi AED	33/227	2.66 (0.94–7.50)	2.67 (0.81–8.77)	Ever use of HDACi AED	27/343	0.50 (0.25–1.00)	0.61 (0.31–1.23)
**Intensity of HDACi AED usage (DDDs per year)**							
1st tertile	10/75	2.36 (0.74–7.52)	3.91 (1.07–14.22)	1st tertile	10/124	0.77 (0.28–2.12)	1.27 (0.46–3.50)
2nd tertile	11/76	2.66 (0.85–8.34)	1.70 (0.46–6.28)	2nd tertile	12/105	0.58 (0.24–1.43)	0.56 (0.22–1.46)
3rd tertile	12/76	2.98 (0.96–9.23)	3.31 (0.91–12.06)	3rd tertile	5/114	0.35 (0.13–0.89)	0.46 (0.18–1.18)
P for trend by tertile		0.002	0.070	P for trend by tertile		0.002	0.048

Study cohort of 9261 prostate cancer cases from the FinRSPC.

^a^Calculated with Cox regression adjusted for age, simultaneous usage of other drugs (NSAIDs, aspirin, anti-diabetic drugs, anti-hypertensive drugs and statins), FinRSPC screening trial arm, Charlson co-morbidity score, prostate cancer risk group and primary treatment of PCa.

### Lag-time analyses

Prostate cancer mortality remained elevated in AED users compared to non-users in a 1-year and 3-year lag-time analyses (Table 4). No difference in prostate cancer mortality between users of HDACi and other AEDs was found in the lag-time analyses.

**Table 4:** Risk of prostate cancer death in a 1-year and 3-year lag-time analyses, when comparing users of AEDs to non-users and users of HDACi AEDs to users of other AEDs.

Risk of prostate cancer death							
Anti-epileptic drug use compared to non-users				Use of HDACi AEDs compared to use of non-HDACi AEDs			
1-year lag-time analysis							
	Number of PCa deaths/PCa cases	HR (95% CI)_age adjusted_	${{{{{\mathbf{HR}}}}}} ({{{{{\mathbf{95\%}}}}}} {{{{{\mathbf{CI}}}}}})_{{{{{{\mathbf{multivariable}}}}}}\; {{{{{\mathbf{adjusted}}}}}}^{\mathbf a}}$		Number of PCa deaths/PCa cases	HR (95% CI)_age adjusted_	${{{{{\mathbf{HR}}}}}} ({{{{{\mathbf{95\%}}}}}} {{{{{\mathbf{CI}}}}}})_{{{{{{\mathbf{multivariable}}}}}}\; {{{{{\mathbf{adjusted}}}}}}^{\mathbf a}}$
No use of AEDs	861/8665	Ref.	Ref.	Use of non-HDACi AEDs	37/318	Ref.	Ref.
Ever use of any AED	59/596	1.56 (1.08–2.24)	1.31 (0.90–1.92)	Ever use of HDACi AEDs	27/343	1.07 (0.48–2.40)	1.11 (0.49–2.53)
**Intensity of usage (DDDs per year)**							
1st tertile	21/183	1.18 (0.44–3.16)	1.50 (0.56–4.01)	1st tertile	10/124	0.23 (0.03–1.82)	0.34 (0.04–2.68)
2nd tertile	22/216	1.93 (1.06–3.49)	1.49 (0.79–2.78)	2nd tertile	12/105	1.46 (0.58–3.70)	1.28 (0.49–3.31)
3rd tertile	16/197	1.48 (0.89–2.46)	1.17 (0.69–1.99)	3rd tertile	5/114	1.18 (0.47–2.92)	1.26 (0.50–3.19)
**3-year lag-time analysis**							
No use of AEDs	861/8665	Ref.	Ref.	Use of non-HDACi AEDs	37/318	Ref.	Ref.
Ever use of any AED	59/596	1.80 (1.29–2.51)	1.55 (1.09–2.20)	Ever use of HDACi AEDs	27/343	1.50 (0.66–3.42)	1.44 (0.62–3.31)
**Intensity of usage (DDDs per year)**							
1st tertile	21/183	1.52 (0.75–3.05)	2.02 (1.01–4.08)	1st tertile	10/124	1.12 (0.38–3.33)	1.43 (0.48–4.27)
2nd tertile	22/216	1.97 (1.11–3.48)	1.43 (0.76–2.67)	2nd tertile	12/105	1.71 (0.66–4.41)	1.28 (0.48–3.44)
3rd tertile	16/197	1.86 (1.13–3.04)	1.45 (0.87–2.43)	3rd tertile	5/114	1.58 (0.62–4.02)	1.59 (0.62–4.12)

Study cohort of 9261 prostate cancer cases from the FinRSPC.

^a^Calculated with Cox regression adjusted for age, simultaneous usage of other drugs (NSAIDs, aspirin, anti-diabetic drugs, anti-hypertensive drugs and statins), FinRSPC screening trial arm, Charlson co-morbidity score, prostate cancer risk group and primary treatment of PCa.

### Sensitivity analysis

In a new-user analysis where men with pre-diagnostic use of AEDs were excluded, users of AEDs had elevated prostate cancer mortality compared to non-users (HR 2.08, 95% CI 1.36–3.19), whereas users of HDACi AEDs had lower prostate cancer mortality than users of other AEDs (HR 0.41, 95% CI 0.17–0.99).

In an analysis where AED use only 2 years prior to PCa diagnosis was taken into account, users of AEDs continued to have elevated PCa mortality compared to non-users of AEDs (multivariable adjusted HR 1.42, 95% CI 0.93–2.18). No difference in PCa mortality in users of HDACi AEDs compared to users of other AEDs was found in this analysis (multivariable adjusted HR 2.95, 95% CI 0.87–9.98).

### Subgroup analyses

**Fig. 1:**
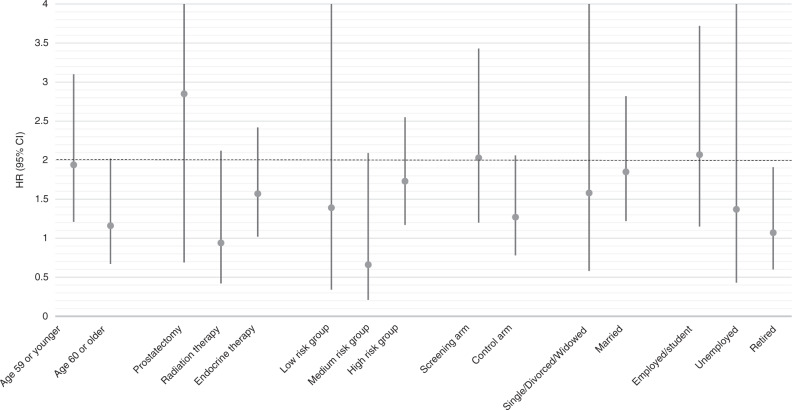
Risk of prostate cancer death by anti-epileptic drug use. Analysis stratified by background characteristics. Study cohort of 9261 prostate cancer cases from the FinRSPC.

**Fig. 2:**
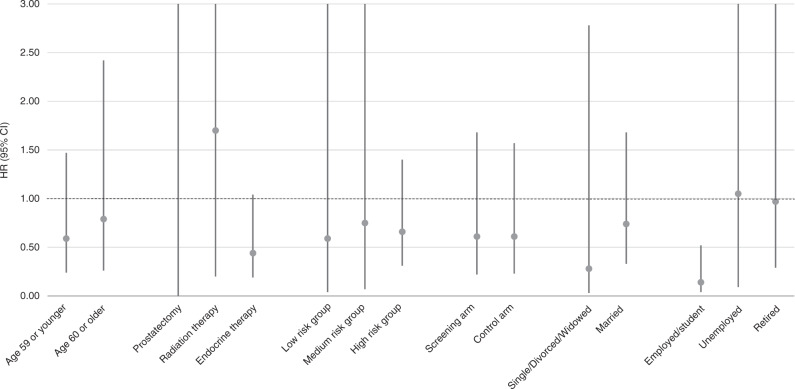
Risk of prostate cancer death by use of anti-epileptic drugs with HDAC inhibitory properties compared to usage of other anti-epileptic drugs. Analysis stratified by background characteristics. Study cohort of 9261 prostate cancer cases from the FinRSPC.

No significant differences in risk related to AED use were found in analyses stratified by age group, primary PCa treatment, PCa risk group, trial arm, marital or socioeconomic status (all *p* > 0.05) (Figs. 1 and 2).

## Discussion

Our study showed that men with prostate cancer using AEDs have higher prostate cancer mortality compared to non-users of AEDs. No clear benefit was seen from either pre- or post-diagnostic use of HDACi AEDs compared to other AEDs. However, when pre-diagnostic use of AEDs was excluded, HDACi AED users had lower prostate cancer mortality compared to users of other AEDs and high-intensity use of HDACi AEDs was associated with a significant decreasing trend in the risk of PCa death.

To our knowledge, this is the first study to comprehensively evaluate the effect of HDACi AED use on prostate cancer mortality. Previous studies have evaluated only HDACi AEDs and prostate cancer risk showing conflicting results. A cohort study of 26,911 US veterans found no association between valproic acid use and prostate cancer risk overall. A Danish population-based case-control study found no decrease in prostate cancer risk among valproic acid users, but their follow-up time was only 5 years, with no more than six exposed cases. A British cohort study of 3000 patients with epilepsy found increased prostate cancer risk among men using valproic acid, but it was based on only eight exposed cases. In our previous study, we found a significant prostate cancer risk reduction among users of valproic acid, carbamazepine and phenobarbital. However, only users of carbamazepine had a significant risk reduction for advanced (lymph-node positive or metastatic) disease. Users of HDACi AEDs had a similar prostate cancer risk compared to users of other AEDs in the FinRSPC study population [18]. In our current study, either pre- or post-diagnostic use of HDACi AEDs was not clearly associated with lower prostate cancer mortality compared to users of other AEDs. The results of lag-time analyses further indicate that the use of HDACi AEDs have no protective effect on prostate cancer mortality in long-term use. In new-user analysis users of HDACi AED had lower PCa mortality compared to users of other AEDs. This finding might be connected to protopathic bias, which affects the choice of AED in men with advanced cancer. However, higher intensity of HDACi AED use was associated with an inverse relation to PCa mortality and we cannot rule out the possibility that higher intensity of HDACi AED use has a protective effect on PCa mortality, a finding that warrants further investigation in future studies.

Epilepsy is associated with increased overall cancer mortality [25–27]. Our finding of increased prostate cancer mortality among users of AEDs in general probably reflects increased cancer mortality among people with epilepsy. However, we did not know the indication for the use of medication, and AEDs are used also for other conditions than epilepsy, which complicates the interpretation of the results. Cancer incidence rates among the users of anti-epileptic drugs, in general, have been reported in several studies [19–21] while studies exploring the effects of anticonvulsants on the risk of cancer death are sparse [28]. Still, it remains uncertain, whether anti-epileptic drugs promote cancer or protect from it. It seems that anticonvulsants do not increase cancer incidence and mortality as much as earlier has been suggested [21, 28]. In our study, we were able to identify a subgroup of men with confirmed epilepsy and found no difference in prostate cancer mortality among them compared to men with no AED use. Within this subgroup no difference was observed between users of HDACi AEDs and users of other AEDs, but the analysis had low statistical power since the sample size was small.

Our study has several strengths. A large population-based cohort was used to evaluate prostate cancer mortality in men using AEDs. Finnish healthcare system is tax-funded, with a minimal role for the private sector. Therefore, all Finns have similar access to healthcare and AEDs regardless of their income or social status. Thus, our study cohort is truly population-based. Accurate information was obtained on prostate cancer cases, major prognostic factors, prostate cancer deaths and anti-epileptic drug use through comprehensive nationwide registers. No recall bias affected the estimation of exposure since the information on drug purchases was obtained from a prescription database. Accurate information on medication purchases allowed analysing usage in a time-dependent manner to minimise immortal time bias. We were also able to evaluate dose-dependence of risk associations, which is not common in pharmacoepidemiologic studies.

Our study also has some limitations. We did not know the indication for drug usage and some AEDs are also used in the management of non-epileptic conditions, such as neuropathic pain and migraine. Drugs administered to hospital inpatients are not recorded in the prescription database, which causes underestimation of the exposure. Also, we did not have information on the actual intake of medication, which might lead to an overestimation of the exposure. Alcohol usage, smoking and physical activity are lifestyle factors that could cause confounding in our results and we did not have information on these. There is growing evidence that physical exercise can lower prostate cancer mortality and that smoking at the time of PCa diagnosis leads to increased prostate cancer mortality. Possible confounding by risk factors would presumably elevate the observed risk among AED users compared to non-users. However, it would not likely explain the difference between HDACi users and non-HDACi users.

To summarise, users of AEDs have increased prostate cancer mortality. Use of HDACi AEDs showed no clear benefit regarding PCa mortality compared to other AEDs in this study, although PCa mortality tended to decrease along with the increasing intensity of HDACi AED use. Further studies are needed to elucidate whether this decreasing risk trend is driven by biology or rather by clinical factors affecting the selection of AEDs in advanced cancer.

## Supplementary information

Supplementary tables

## Data Availability

The datasets generated during and/or analysed during the current study are available from the corresponding author on reasonable request.
