# Supplementary material for: Anti-epileptic drugs and prostate cancer-specific mortality compared to non-users of anti-epileptic drugs in the Finnish Randomized Study of Screening for Prostate Cancer
Source: Br J Cancer. 2022 May 3;127(4):704–11. doi: 10.1038/s41416-022-01817-3 (PMC9381528; doi:10.1038/s41416-022-01817-3)
Supplement: Supplementary file 1 — Supplementary tables [file 41416_2022_1817_MOESM1_ESM.docx]

**Supplementary table 1.** Risk of prostate cancer death by HDACi antiepileptic drug use. Study cohort of 9,261 prostate cancer cases from the FinRSPC.

| **Risk of prostate cancer death** | | | | | | | | |
| --- | --- | --- | --- | --- | --- | --- | --- | --- |
|  | **AED use before diagnosis** | | |  |  | **AED use after diagnosis** | | |
|  | Number of PCa deaths/PCa cases | HR (95 % CI) _age adjusted_ | HR (95 % CI) _multivariable adjusted*_ |  |  | Number of PCa deaths/PCa cases | HR (95 % CI) _age adjusted_ | HR (95 % CI) _multivariable adjusted*_ |
| No AED use | 883/8,965 | Ref | Ref |  | No AED use | 861/8,665 | Ref | Ref |
| Ever use of HDACi AEDs | 33/227 | 2.02 (1.43 – 2.86) | 1.62 (1.11 – 2.35) |  | Ever use of HDACi AEDs | 27/343 | 1.31 (0.82 – 2.10) | 1.15 (0.71 – 1.87) |
| **Intensity of usage (DDDs per year)** | | | | | | | | |
| 1^st^ tertile | 10/75 | 1.79 (0.96 – 3.34) | 2.37 (1.26 – 4.43) |  | 1^st^ tertile | 10/124 | 1.49 (0.77 – 2.87) | 2.10 (1.08 – 4.06) |
| 2^nd^ tertile | 11/76 | 2.02 (1.11 – 3.66) | 1.03 (0.53 – 1.99) |  | 2^nd^ tertile | 12/105 | 1.86 (1.00 – 3.47) | 1.18 (0.59 – 2.37) |
| 3^rd^ tertile | 12/76 | 2.26 (1.28 – 4.00) | 2.01 (1.07 – 3.75) |  | 3^rd^ tertile | 5/114 | 0.97 (0.46 – 2.03) | 0.90 (0.43 – 1.90) |

* Calculated with Cox regression adjusted for age, simultaneous usage of other drugs (NSAIDs, aspirin, antidiabetic drugs, antihypertensive drugs, and statins), FinRSPC screening trial arm, Charlson co-morbidity score, prostate cancer risk group and primary treatment of PCa.

**Supplementary table 2.** Risk of prostate cancer death by valproic acid (VPA) use compared to non-users of AEDs and compared to users of AEDs without HDACi properties. Study cohort of 9,261 prostate cancer cases from the FinRSPC.

| **Risk of prostate cancer death** | | | | | | | | | |
| --- | --- | --- | --- | --- | --- | --- | --- | --- | --- |
|  | **VPA use before diagnosis** | | |  |  | **VPA use after diagnosis** | | | |
|  | Number of PCa deaths/PCa cases | HR (95 % CI) _age adjusted_ | HR (95 % CI) _multivariable adjusted*_ |  |  | Number of PCa deaths/PCa cases | HR (95 % CI) _age adjusted_ | HR (95 % CI) _multivariable adjusted*_ |  |
| No AED use | 883/8,965 | Ref | Ref |  | No AED use | 861/8,665 | Ref | Ref |  |
| Ever use of VPA | 8/53 | 2.53 (1.26 – 5.07) | 1.29 (0.58 – 2.89) |  | Ever use of VPA | 9/133 | 1.82 (0.91 – 3.66) | 1.25 (0.59 – 2.63) |  |
| **Risk of prostate cancer death** | | | | | | | | | |
|  | Number of PCa deaths/PCa cases | HR (95 % CI) _age adjusted_ | HR (95 % CI) _multivariable adjusted_ |  |  | Number of PCa deaths/PCa cases | HR (95 % CI) _age adjusted_ | HR (95 % CI) _multivariable adjusted_ |  |
| Ever use of non-HDACi AED | 8/112 | Ref | Ref |  | Ever use of non-HDACi AED | 37/318 | Ref | Ref |  |
| Ever use of VPA | 8/53 | 3.37 (1.02 – 11.21) | 2.16 (0.54 – 8.65) |  | Ever use of VPA | 9/133 | 0.70 (0.30 – 1.64) | 0.67 (0.27 – 1.63) |  |

* Calculated with Cox regression adjusted for age, simultaneous usage of other drugs (NSAIDs, aspirin, antidiabetic drugs, antihypertensive drugs, and statins), FinRSPC screening trial arm, Charlson co-morbidity score, prostate cancer risk group and primary treatment of PCa.

**Supplementary table 3.** Population characteristics of men having used AEDs only after diagnosis of PCa. Study cohort of 9,261 prostate cancer cases from the FinRSPC.

|  | **Antiepileptic drug use** | |
| --- | --- | --- |
|  | AED use | HDACi AED use |
| **N of prostate cancer cases** | 444 | 223 |
| **N of deaths** | 194 (43.7 %) | 107 (48.0 %) |
| **N of prostate cancer deaths** | 41 (9.2 %) | 12 (5.4 %) |
| **N of 1) low**  **2) medium**  **3) high risk PCa** | 1) 157 (35.4 %)  2) 174 (39.2 %)  3) 110 (24.8 %) | 1) 84 (37.7 %)  2) 89 (39.9 %)  3) 50 (22.4 %) |
| **Median age at diagnosis** | 68 | 68 |
| **Screening arm** | 178 (40.1 %) | 86 (38.6 %) |
| antidiabetic drugs; n (%) | 75 (16.9 %) | 35 (15.7 %) |
| statins; n (%) | 256 (57.7 %) | 128 (57.4 %) |
| antihypertensive drugs; n (%) | 374 (84.2%) | 184 (82.5 %) |
| aspirin; n (%) | 86 (19.4 %) | 49 (22.0 %) |
| NSAIDs; n (%) | 409 (92.1 %) | 204 (91.5 %) |
| Active surveillance/watchful waiting | 90 (20.3 %) | 50 (22.4 %) |
| Radical prostatectomy | 96 (21.6 %) | 47 (21.1 %) |
| Radiation therapy | 176 (39.6 %) | 98 (43.9 %) |
| Endocrine therapy | 79 (17.8 %) | 26 (11.7 %) |
| Palliative | 0 (0.0 %) | 0 (0.0 %) |
| Other | 3 (0.7 %) | 2 (0.9 %) |
